# Coparenting Alleviated the Effect of Psychological Distress on Parental Psychological Flexibility

**DOI:** 10.3389/fpsyg.2021.646380

**Published:** 2021-07-16

**Authors:** Yongju Yu, Yan Xiao

**Affiliations:** Department of Social Work, School of Sociology and Law, Sichuan International Studies University, Chongqing, China

**Keywords:** anxiety, depression, coparenting, parental psychological flexibility, Chinese parents

## Abstract

Parenting is full of challenges and responsibilities. It is particularly important for parents to be open to parental difficult experiences and adopt behaviors consistent with self-chosen values, which termed as parental psychological flexibility (PPF). However, few studies have focused on the effect of psychological distress (anxiety and depression) on different components of PPF. This study examined the effect of psychological distress on the three components of PPF (cognitive defusion, committed action, and acceptance) as well as the role of coparenting quality in Chinese parents. A total of 462 parents of children aged 1–18 years completed self-report measures of anxiety, depression, coparenting, and PPF. Our results revealed that higher level of PPF went along with less anxiety and depression, while it was also associated with better coparenting quality. Coparenting partially mediated the effect of anxiety on cognitive defusion and acceptance and fully mediated the effect of depression on cognitive defusion and acceptance. Moderation analyses showed that the link between anxiety and cognitive defusion, as well as the link between anxiety and acceptance were moderated by coparenting. We discussed the implications of coparenting as a protective factor in alleviating the negative effect of psychological distress on PPF.

## Introduction

No matter which stage your child is in, parenting is full of challenges and responsibilities (Moyer and Sandoz, 2015). New parents may have to learn skills such as how to balance discipline and overcontrol, to teach children basic life skills, as well as to help children improve their social adaptability. Parents of school-age children need to teach their children how to deal with the pressure from learning and peer competition, perplexity in puberty, confusion of self-identity, etc. These experiences bring pain and happiness simultaneously to individuals who are parents. The notion of “parental psychological flexibility (PPF)” provides a new perspective for us to research on parenting.

Parental psychological flexibility is defined as parents’ accepting negative thoughts, emotions and urges about one’s child and still acting in ways that are consistent with effective parenting (Burke and Moore, 2015). PPF can be measured by the Parental psychological flexibility Questionnaire (PPFQ) developed by Burke and Moore (2015). It consists of three factors: cognitive defusion, committed action, and acceptance. Cognitive defusion refers to the ability to separate thoughts, emotions, physical sensations, and urges from the evaluation of specific events and to select behaviors that are likely to be effective for their context. Committed action refers to the flexible responses based on specific circumstances and personal values. Acceptance is defined as the willingness to experience individual events without changing the frequency or form of events (Burke and Moore, 2015).

Previous studies mainly focus on the positive impact of PPF on child functioning, such as chronic pain (Wallace et al., 2016) and mental health (Teetsel et al., 2014). Little research has been performed to test the impacts of internal and external factors on PPF. Nevertheless, the process model and the family systems theory provide a theoretical perspective for the research of this topic. According to the process model, parental functioning is multiply determined and contextual support and individual psychological well-being can affect parenting (Belsky, 1984). The family systems theory proposed that marital and parent-child relationships are interrelated (Cox and Paley, 2003) and studying the interactions of family members can better illustrate the process of parenting. Accordingly, parents’ psychological distress (anxiety and depression), the support and interaction between fathers and mothers in child rearing (termed as coparenting) were studied emphatically in this study.

Emotional distress has been demonstrated to lead parents to use ineffective parenting methods (Bayer et al., 2006). Parents may lack concerns for their children due to excessive involvement in negative experiences, over interfere with their children or adopt inappropriate parenting practice due to constant worry (Moyer and Sandoz, 2015). Anxiety symptoms often lead to less nurturing and more restrictions (Lindhout et al., 2006; Moyer and Sandoz, 2015), while depression symptoms are associated with more negative physical behaviors (Querido et al., 2001) and less verbal communication (Coyne et al., 2007). A recent study demonstrated that anxiety and depression have significant and negative impacts on parenting behaviors and practices (Moreira et al., 2019). Parents with more depression and anxiety symptoms had a stronger tendency to adopt psychological aggression to discipline their children (Wang et al., 2019). Unfortunately, there is still a major gap in our knowledge about the impact of psychological distress on PPF.

According to the family systems theory, the functioning and well-being of a family member depend on the interactions among each one of the whole family (Minuchin, 1974, 1988). As the executive subsystem of the family, coparenting reflects mutual support and coordinate between husband and wife in their roles as parents (Feinberg et al., 2016). Coparenting has been demonstrated to be closely related to family function, parental rearing patterns, and child development (Metz et al., 2018a). Schoppe-Sullivan et al. (2016) reported that mothers’ perceptions of stronger supportive coparenting were associated with less parenting stress when parenting self-efficacy was low. It was also found that the severity of parental anxiety was associated with more destructive coparenting, which in turn was related to children’s fearful temperament (Metz et al., 2018b). Coparenting quality can easily spill over into the parent-child relationship. A longitudinal study on 69 parental couples revealed that coparenting mediated the relationships between maternal depression symptoms and child symptoms (Tissot et al., 2016). Another investigation on 182 families showed that maternal coparenting attitudes could predict fathers’ active participation (Yan et al., 2018). Such findings suggest the necessity of exploring the mediating/moderating role of coparenting in the relationship between psychological distress and parenting quality. Scrimgeour et al. (2013) found that supportive coparenting may enhance the benefits of positive parenting and buffer the risks of negative parenting on children’s prosocial behaviors. Conversely, coparenting conflicts may overwhelm parents’ self-management and undermine their ability as sensitive caregivers of their adolescents (Martin et al., 2017). Therefore, the family process model linking parental psychological distress with their PPF was tested in this study. We assume that parents’ anxiety and depression are related to a decrease in PPF, which will be regulated by coparenting.

Accordingly, the current study sought to clarify the relationship between psychological distress (i.e., anxiety and depression) and PPF, as well as to test the role of coparenting in Chinese parents of children 1–18 years old. The research hypotheses are as follows:

H1: Less anxiety and depression are related to higher level of PPF.

H2: Less anxiety and depression are related to better coparenting quality.

H3: Better coparenting is associated with higher level of PPF.

H4: Coparenting mediates the link between anxiety, depression, and PPF.

H5: Coparenting serves as a moderator between anxiety, depression, and PPF.

## Materials and Methods

### Participants and Procedures

The current study was approved by the Ethics Committee of Sichuan International Studies University (IRB number: 20200001). All procedures were carried out in accordance with the Declaration of Helsinki. Through online advertisements and the We Chat friends circle, by convenient sampling, 490 parents who had at least one child aged 1–18 years old were recruited from four communities in Chongqing city, China. They completed online self-reported measures of their background information, anxiety, depression, coparenting, and PPF. Nine participants were excluded since their time to fill in the questionnaire was less than 300 s. Besides that, seven single parents and 12 divorced parents were excluded. In the final sample, there were 462 participants (114 fathers and 348 mothers) aged from 20 to 52 years (mean = 36.43, SD = 6.18) and their children aged from 1 to 18 years (mean = 8.15, SD = 5.17). Among these parents, 71 (15.4%) were educated up to less than high school, 63 (13.6%) had completed high school, 224 (48.5%) had junior college or bachelor’s degrees, and 104 (22.5%) had master’s degrees or above. Of the participants, 310 had only one child, 152 had two or more children. Additionally, there were 159 parents of toddlers and preschool children (1–5 years old), 98 parents of primary children (6–11 years old), 119 parents of adolescents (12–18 years old), and 86 parents having two or more children at different stages.

Prior to filling in the questionnaire, researchers explained the purpose and contents of this study to all participants. Participants were told that their anonymity and confidentiality would be maintained. Moreover, participants had access to their own results and corresponding explanations as soon as they completed the questionnaire.

### Study Measures

#### Anxiety

The anxiety symptoms of parents over the past 2 weeks were assessed by the seven-item Generalized Anxiety Disorder Questionnaire (GAD-7) (Spitzer et al., 2006; Qu and Sheng, 2015). For example, “Not being able to stop or control worrying”. Each item was rated on a 4-point Likert scale ranging from 0 (not at all) to 3 (nearly every day). According to Qu and Sheng (2015), the GAD-7 has good psychometric properties in Chinese population. The Cronbach’s alpha for GAD-7 was 0.915 in this study.

#### Depression

The depression symptoms in the past 2 weeks were assessed by the 9-item Patient Health Questionnaire (PHQ-9) (Kroenke et al., 2001; Lai et al., 2010; Zhang et al., 2013). For example, “Thoughts that you would be better off dead or of hurting yourself in some way.” Each item was rated on a 4-point Likert scale ranging from 0 (not at all) to 3 (nearly every day). The higher the score, the more serious the depression is. The PHQ-9 has good reliability and validity in Chinese samples (Lai et al., 2010; Zhang et al., 2013). The Cronbach’s alpha for PHQ-9 was 0.895 in our sample.

#### Coparenting Quality

Coparenting relationship quality was assess by using the 14-item Coparenting Relationship Scale (CRS, Feinberg et al., 2012; Wu et al., 2017). For example, “We are growing and maturing together through experiences as parents.” Each item was rated on a 0–6 Likert scale. This scale has been confirmed to possess good psychometric properties in Chinese parents (Wu et al., 2017). The Cronbach’s alpha for the total scale was 0.864 in our sample.

#### Parental Psychological Flexibility

Psychological flexibility among parents was assessed by the 19-item PPFQ (Burke and Moore, 2015; Li et al., 2018). For example, “My emotions get in the way of the being the type of parent I would ideally like to be.” It comprises three factors: cognitive defusion, committed action, and acceptance. Respondents were asked to rate all items from 1 (never true) to 7 (always true). Chinese version of PPFQ has been proved good reliability and validity (Li et al., 2018). The Cronbach’s alpha values for the cognitive defusion, committed action, and acceptance subscales in our sample were 0.869, 0.718, and 0.815, respectively. Additionally, the Cronbachy, alpha value for the total scale was 0.880 in this study.

### Data Analysis

Independent sample *t*-test and one-way ANOVA were carried out to compare the differences of main study variables in gender, number of children, education level, and developmental stage of children. Pearson correlation analyses were performed to describe the associations of study variables and to test initial hypotheses H1, H2, and H3. Structural equation modeling (SEM) was conducted to assess the mediating role of coparenting in the relationships of anxiety, depression, and PPF (hypothesis H4). Hierarchical multiple regression analyses were conducted to examine whether coparenting moderates the associations between anxiety, depression, and PPF (hypothesis H5). *P* < 0.05 indicates statistical significance in the current study. SPSS 24.0 and Amos 18.0 were used for data analyses.

## Results

### Preliminary Analyses

Table 1 presents the results of descriptive statistics and correlation analyses. It was found that higher scores of three subscales of PPF (cognitive defusion, committed action, and acceptance) went along with less anxiety and depression, while they were related to better coparenting (all *P*s < 0.01). Meanwhile, anxiety and depression were negatively associated with coparenting (all *P*s < 0.01). Therefore, our initial hypotheses H1, H2, and H3 were well supported.

**TABLE 1:** Correlations, means, and standard deviations for main study variables.

	**Possible range**	***Mean* (*SD*)**	**Anxiety**	**Depression**	**Coparenting**
Anxiety	0–21	5.86 (4.77)	–		
Depression	0–27	6.75 (5.29)	0.799**	–	
Coparenting	0–84	56.88 (14.29)	–0.443**	–0.425**	–
Parental psychological flexibility	19–133	88.66 (18.00)	–0.537**	–0.473**	0.395**
Cognitive defusion	8–56	35.67 (10.45)	–0.490**	–0.421**	0.363**
Committed action	5–35	20.00 (6.06)	–0.338**	–0.295**	0.129**
Acceptance	6–42	32.99 (6.16)	–0.406**	–0.377**	0.411**

*Note* **P < 0.01; *n* = 462.

### Effects of Gender, Education, Number of Children, and Developmental Stage of Children

Independent sample *t*-test was perfomed to examine the impacts of gender and number of children on anxiety, depression, coparenting, and PPF. No significant difference was found between fathers and mothers in anxiety, depression, coparenting, and PPF (*P*s > 0.05). Results also revealed that scores of anxiety, depression, coparenting, and PPF for parents with one child were not significantly different from those who had two or more children (*P*s > 0.05).

In order to examine whether there are differences in study variables among parents of children at different stages, parents were divided into four groups: parents of toddlers and preschool children (1–5 years old), parents of primary children (6–11 years old), parents of adolescents (12–18 years old), and parents having two or more children at different stages. The results of one-way ANOVA are described in Table 2. There was no significant group difference in anxiety, depression, and coparenting. However, a significant difference was found in the scores of PPF between groups (*F* = 5.82, *P* < 0.01). LSD *post hoc* tests showed that parents of toddlers and preschool children reported higher levels of PPF than those of other parents (*P*s < 0.05). No difference was found among the other three groups (*P*s > 0.05).

**TABLE 2:** Comparisons in main study variables among parents with child(ren) at different stages.

	**Group 1 (*n* = 159)**	**Group 2 (*n* = 98)**	**Group 3 (*n* = 119)**	**Group 4 (*n* = 86)**	***F***	**Significance**
Anxiety	5.89 ± 4.76	6.12 ± 4.99	5.96 ± 4.64	5.36 ± 4.78	0.43	None
Depression	7.07 ± 5.27	6.57 ± 5.06	6.62 ± 5.28	6.56 ± 5.67	0.29	None
Coparenting	58.55 ± 14.25	54.55 ± 14.56	56.73 ± 14.29	56.64 ± 13.88	1.61	None
Parental psychological flexibility	92.71 ± 17.81	87.86 ± 17.66	85.98 ± 18.01	85.79 ± 17.66	4.51**	Group 1 > Group 2; Group 1 > Group 3; Group 1 > Group 4

*Note* **P < 0.01. Group 1, parents of toddlers and preschool children (1–5 years old); Group 2, parents of primary children (6–11 years old); Group 3, parents of adolescents (12–18 years old); Group 4, parents having two or more children at different stages.

As listed in Table 3, one-way ANOVA also showed that the effect of education level on PPF was significant (*F* = 11.32, *P* < 0.01). LSD *post hoc* tests showed that scores of PPF for parents with education level less than high school were lower than those of other three groups, while scores of PPF for parents with master’s degrees or above were higher than those of other three groups. Besides that, levels of depression were significantly higher for parents who completed high school or below than parents who had master’s degrees or above (*P*s < 0.05).

**TABLE 3:** Comparisons in main study variables among parents with different educational levels.

	**Group A (*n* = 71)**	**Group B (*n* = 63)**	**Group C (*n* = 224)**	**Group D (*n* = 104)**	***F***	**Significance**
Anxiety	6.86 ± 5.26	6.62 ± 4.65	5.62 ± 4.59	5.22 ± 4.78	2.40	None
Depression	7.73 ± 6.10	7.63 ± 5.28	6.67 ± 4.94	5.73 ± 5.31	2.74*	A > D; B > D
Coparenting	53.41 ± 13.87	55.48 ± 14.59	57.98 ± 13.99	57.72 ± 14.76	2.18	None
Parental psychological flexibility	79.37 ± 17.56	86.37 ± 14.23	89.48 ± 16.92	94.63 ± 19.97	11.32**	A < B; A < C; A < D; B < D; C < D

*Note* *P < 0.05; **P < 0.01. A, less than high school; B, high school education; C, junior college or bachelor’s degree; D, master’s degree or above.

### Mediation Analyses

**FIGURE 1:**
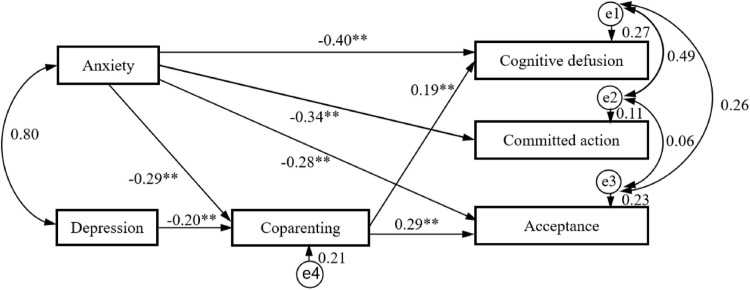
Results of mediation path analysis showing the relationships between anxiety, depression, and parental psychological flexibility with coparenting as a mediator. ^∗∗^*P* < 0.01.

Structural equation modeling was performed by AMOS 18.0 to explore the mediating role in the associations of anxiety, depression, and PPF. In the original model of Figure 1, four pathways did not reach significance (depression → cognitive defusion: *b* = –0.10, *P* = 0.47; depression → committed action: *b* = –0.09, *P* = 0.31; depression → acceptance: *b* = –0.11, *P* = 0.18; and coparenting → committed action: *b* = –0.01, *P* = 0.51). Therefore, we deleted these four non-significant pathways individually. After recalculation, the modified model (Figure 1) revealed a good model fit: χ^2^(4) = 2.980, *P* = 0.40, CFI = 1.000, TLI = 1.000, GFI = 0.998, SRMR = 0.011, and RMSEA < 0.001. It explained 21.0% of coparenting variance, 26.9% of cognitive defusion variance, 11.4% of committed action variance, and 23.2% of acceptance variance. In this model, the results indicated that anxiety had direct negative impacts on cognitive defusion (*b* = –0.40, *P* < 0.01), committed action (*b* = –0.34, *P* < 0.01), and acceptance (*b* = –0.28, *P* < 0.01). Anxiety also exerted indirect negative impacts on cognitive defusion (*b* = –0.06, *P* < 0.01) and acceptance (*b* = –0.08, *P* < 0.01) through coparenting. Moreover, depression only had indirect negative effects on cognitive defusion (*b* = –0.04, *P* < 0.01) and acceptance (*b* = –0.06, *P* < 0.01) through coparenting.

According to previous studies (MacKinnon et al., 2004; Cheong and MacKinnon, 2012), bootstrapping procedures via Amos 18.0 (*k* = 2,000) were carried out to test the significance of the indirect effects. As indicated in Table 4, both anxiety and depression exerted significantly indirect impacts on cognitive defusion and acceptance through coparenting. Sum up, coparenting partially mediated the impacts of anxiety on cognitive defusion and acceptance, while coparenting fully carried the impacts of depression on cognitive defusion and acceptance, rather than committed action. Therefore, the hypothesis H4 that coparenting mediates the link between anxiety, depression, and PPF was partially supported.

**TABLE 4:** Bootstrapping indirect effects and 95% confidence intervals (CI) for the final mediational model.

**Model pathways**	**Point estimates**	**95% CI**	
		**Lower**	**Upper**
Anxiety -> Coparenting -> Cognitive defusion	–0.12	–0.19	–0.06
Anxiety -> Coparenting -> Acceptance	–0.11	–0.16	–0.06
Depression -> Coparenting -> Cognitive defusion	–0.08	–0.13	–0.03
Depression -> Coparenting -> Acceptance	–0.07	–0.12	–0.02

### Moderation Analyses

According to SEM analysis results, anxiety, rather than depression, negatively predicted cognitive defusion, committed action, and acceptance. Therefore, we performed hierarchical linear regressions to test the moderating role of coparenting in the relationship between anxiety and PPF (cognitive defusion, committed action, and acceptance, respectively). All continuous variables were centered. Cognitive defusion was included in the regression model as the dependent variable, while covariates (age, gender, number of children, and education level) were entered into the regression model firstly. Anxiety, depression, and coparenting were included in the model subsequently. Interaction term of anxiety × coparenting was included finally. Table 5 presents the results of hierarchical regression. In line with the findings of mediating analysis, these results showed that anxiety and coparenting, rather than depression, significantly predicted cognitive defusion. There was a significant anxiety × coparenting interaction (β = –0.40, *t* = –3.34, *P* < 0.01), suggesting that coparenting moderated the association between anxiety and cognitive defusion. Same procedures were carried out for committed action and acceptance. Results for acceptance also revealed that there were significant main effects of anxiety and coparenting, rather than depression. Similarly, the anxiety × coparenting interaction significantly predicted acceptance (β = –0.32, *t* = –2.56, *P* < 0.05), suggesting that coparenting moderated the relationship between anxiety and acceptance. However, coparenting did not moderate the relationship between anxiety and committed action (β = –0.20, *t* = –1.49, *P* > 0.05).

**TABLE 5:** The regression models for cognitive defusion and acceptance (beta, standardized regression coefficient).

		**Cognitive defusion**	**Committed action**	**Acceptance**
Step 1	Age	–0.03	0.02	–0.02
	Gender	–0.07	0.02	–0.05
	Number of children	0.01	–0.01	–0.01
	Education level	0.22**	0.24**	0.13*
Step 2	Age	–0.04	<0.01	–0.01
	Gender	–0.08*	<0.01	–0.05
	Number of children	–0.03	–0.03	–0.05
	Education level	0.15**	0.19**	0.06
	Anxiety	–0.37**	–0.29**	–0.21**
	Depression	–0.05	–0.06	–0.09
	Coparenting	0.16**	–0.04	0.27**
Step 3	Age	–0.05	–0.01	–0.03
	Gender	–0.08	0.01	–0.05
	Number of children	–0.04	–0.03	–0.05
	Education level	0.14**	0.19**	0.05
	Anxiety	0.08	–0.07	0.15
	Depression	–0.08	–0.08	–0.12
	Coparenting	0.32**	0.04	0.40**
	Anxiety × coparenting	–0.40**	–0.20	–0.32*
	Step 1	*F* = 7.188; *P* < 0.001; *R*_1_^2^ = 0.243	*F* = 6.823; *P* < 0.001; *R*_1_^2^ = 0.056	*F* = 2.529; *P* = 0.040; *R*_1_^2^ = 0.147
	Step 2	*F* = 28.290; *P* < 0.001; *R*_2_^2^ = 0.551	*F* = 11.977; *P* < 0.001; *R*_2_^2^ = 0.156	*F* = 21.051 *P* < 0.001; *R*_2_^2^ = 0.495
	Step 3	*F* = 26.702; *P* < 0.001; *R*_3_^2^ = 0.566	*F* = 10.787; *P* < 0.001; *R*_3_^2^ = 0.160	*F* = 18.465; *P* < 0.001; *R*_3_^2^ = 0.506
	Δ*R*^2^ (Step 3 - Step 2)	0.015	0.004	0.011

*Note n* = 462; *P < 0.05; **P < 0.01.

**FIGURE 2:**
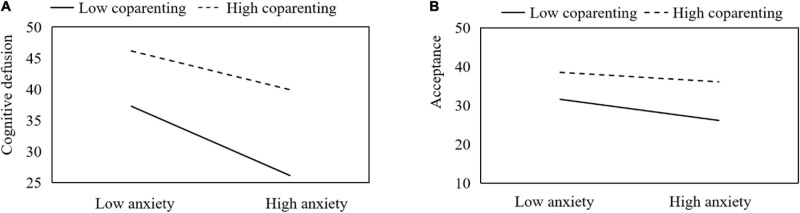
Simple slopes of anxiety predicting cognitive defusion **(A)** and acceptance **(B)** at high (+1 SD) and low (–1 SD) levels of coparenting.

According to Holmbeck’s suggestion (Holmbeck, 2002), we calculated the simple slopes at 1 SD above (>71.17) and below (<42.59) the mean coparenting level to test the impact of the anxiety × coparenting interaction on cognitive defusion, and the impact of the anxiety × coparenting interaction on acceptance. When coparenting reported by parents was low, the link between anxiety and cognitive defusion was stronger (β = –0.56, *t* = –5.30, *P* < 0.01) as compared to the case when coparenting was high (β = –0.45, *t* = –4.31, *P* < 0.01). Figure 2A showed that the link between anxiety and cognitive defusion was more stronger for parents who had poorer coparenting quality compared with those reporting better coparenting quality. Figure 2B revealed that the link between anxiety and acceptance was more significant (β = –0.24, *t* = –2.13, *P* = 0.03 < 0.05) for participants who had poorer coparenting quality, while the link between anxiety and acceptance was not significant for parents reporting higher levels of coparenting (β = –0.17, *t* = –1.51, *P* = 0.14 > 0.05). These findings partially supported the hypothesis H5 that coparenting moderates the relationship between anxiety, depression, and PPF.

## Discussion

The results suggested that total scores of PPF in this sample were lower than those in a previous study based on 1,075 parents of primary children (88.66 ± 18.00 vs. 96.80 ± 15.60, *t* = −9.72, *P* < 0.01; Li et al., 2018). This revealed that the protective and risk factors of PPF should be explored in order to develop psychological intervention measures for the sake of facilitating their PPF. In line with previous studies (Li et al., 2018; Li, 2019), the educational level had a significant impact on PPF. In particular, parents with education level less than high school had the lowest level of PPF, while those who had master’s degrees or above reported the highest level of PPF. No significant difference was found between another two groups. The possible explanation underlying this phenomenon is that the education level of parents may act as a proxy for other factors such as family income, social status, coping style, etc. (Bluth et al., 2020). The reason behind this phenomenon may be that parents of toddlers and preschool children faced relatively simple parenting matters, so they reported a higher level of self-perceived PPF. With the growth of children, the problems in parenting increased. Parents should not only take care of children’s daily life, but also teach their children how to get along with others, how to take social responsibility, how to cope with academic pressure and competitive environment (Quach et al., 2015). Therefore, they reported a lower level of self-perceived PPF. It requires our more attention in order to improve the PPF of parents of school-age children.

Our findings clearly supported the hypothesis that anxiety and depression are negatively associated with PPF. In agreement with previous studies (Moyer and Sandoz, 2015; Sairanen et al., 2018), emotional distress, such as anxiety and depression, increased the risk for psychological inflexibility in the context of parenting. Results of SEM showed that anxiety exhibited direct impacts on three factors of PPF and indirect impacts on cognitive defusion and acceptance through coparenting. It further confirmed that parents’ anxiety may hinder the development of their adaptive parenting skills, thereby leading to: “anxiety-enhancing” parental behaviors, such as denial and rejection (Ginsburg and Schlossberg, 2002; Hudson and Rapee, 2002), which reflects the core characteristics of parental psychological inflexibility. By contrast, depression only had indirect impacts on cognitive defusion and acceptance via coparenting. As mentioned by Vćver et al. (2015), parents in depression state do not pay enough attention to children’s psychological needs and feelings, and rarely carry out continuous social interaction with their children. This phenomenon may be due to the fact that parents’ depression symptoms are often accompanied by cognitive impairments (Pettersen and Albers, 2001), which in turn, contribute to less responsive and less positive parenting behaviors toward their children (Kim-Cohen et al., 2005). However, these withdrawal behaviors cannot be well captured in the PPFQ. Accordingly, it is understandable that anxiety exhibited a stronger negative impact on PPF than depression in this study. What’s even more concerning, as proposed by previous studies (Majdandžić et al., 2012; Metz et al., 2018b; Williams, 2018), anxiety and depression symptoms play a role in the undermining coparenting behavior of parents. The possible explanation might be that anxiety and depression lead to more couple conflicts, inconsistent parenting, and unreasonable division of labor (Lamela et al., 2016), thereby resulting in poor coparenting quality.

This study also sought to examine the impact of coparenting quality on PPF, as well as its mediating and moderating roles. As expected, it was found that coparenting quality significantly predicted cognitive defusion and acceptance, rather than committed action, which indicated that coparenting could help parents avoid passivity, severity and boredom, increase the possibility of perceiving and strengthening children’s positive behaviors, and make parents’ behaviors consistent with their parenting values. The results actually explained the finding that the support and coordination between couples can promote family function, therefore they tend to adopt more effective parental strategies (Sotomayor-Peterson et al., 2013). Moreover, the moderation model showed that better coparenting quality moderated the negative impact of anxiety on cognitive defusion and acceptance. That is, relative to parents with poorer coparenting quality, those who had better coparenting quality were more likely to accept children’s and their own psychological distress and thoughts and less likely to be disturbed by anxiety. This study shed light on the associations of anxiety, depression, coparenting quality, and PPF. Our results revealed the fact that coparenting acts as a protective factor for alleviating the impact of parental anxiety on PPF. These findings supported the process model (Belsky, 1984) and the family systems theory (Cox and Paley, 2003) by suggesting that PPF is multiply determined and individual mental health and parenting support and interaction between husband and wife can affect parenting quality.

Nevertheless, several shortcomings of this study should be noted. Firstly, the cross-sectional design limited its ability to infer the causal relationship between anxiety, depression, coparenting, and PPF. Besides that, the moderating and mediating roles of the same construct were tested simultaneously in this study. Although this method is often used by other researchers (Hayes, 2013; Güngr and Uman, 2020). Karazsia and Berlin (2018) proposed a more robust model and pointed out that a variable can serve as both a mediator and a moderator, but at different time points within the same model. Therefore, a longitudinal study should be carried out in the future in order to accurately capture the role of coparenting between psychological distress and PPF. Secondly, the application of self-reported measures affected the objectivity of this study to a certain extent. Some other assessment methods (e.g., peer-reports and objective outcomes) should be used to avoid the possible effects of social expectations. Finally, we failed to collect some demographic information such as family income, parental stress, children’s internalizing and externalizing behaviors which may be related to PPF.

Despite these limitations, current findings demonstrated that psychological distress, especially anxiety, had significant and negative impacts on parental flexibility. In addition, coparenting played a vital role between psychological distress and PPF. On the one hand, anxiety and depression can affect PPF by lessening the quality of coparenting. On the other hand, good coparenting quality can alleviate the impact of anxiety on PPF. These findings have significant implications for parental practice and research by suggesting that coparenting may serve as a potential intervention target for enhancing PPF. Our results also suggested that parents with low educational background and parents of school-age children should be investigated deeply in future studies. Regarding parents with low education background, social support and positive empowerment may be important ways to improve their parental flexibility. More attention should be paid to the parenting pressure faced by this vulnerable group, so as to formulate targeted solutions.

## Data Availability Statement

The raw data supporting the conclusions of this article will be made available by the authors, without undue reservation.

## Ethics Statement

The studies involving human participants were reviewed and approved by the Ethics Committee of Sichuan International Studies University. The patients/participants provided their written informed consent to participate in this study.

## Author Contributions

YY conceived and designed the investigation, analyzed the data, and wrote the article. YX collected the data and revised the manuscript. Both authors contributed to the article and approved the submitted version.

## Conflict of Interest

The authors declare that the research was conducted in the absence of any commercial or financial relationships that could be construed as a potential conflict of interest.
